# Investigating how immersive virtual reality and active navigation mediate the experience of virtual concerts

**DOI:** 10.1038/s41598-023-35369-0

**Published:** 2023-05-25

**Authors:** Shih-Yu Lo, Chih-Yuan Lai

**Affiliations:** Institute of Communication Studies, National Yang Ming Chiao Tung University, Hsinchu, Taiwan

**Keywords:** Neuroscience, Psychology

## Abstract

We conducted an experiment to examine how virtual reality (VR) and active navigation interact to improve audience experience in virtual concerts. To manipulate the medium, the participants were presented with concert-related audiovisual stimuli via a head-mounted VR device or a computer. To manipulate the participants’ access to different perspectives (navigation mode), they were allowed to actively switch, or were passively guided, between the audience’s perspective and the performer’s perspective. According to the results, VR and active navigation induced a higher sense of presence (feeling of being somewhere else) than did computer and passive navigation, and thus, they increased the audience’s state of flow and gave them higher degrees of satisfaction and concert-attending intention. VR and active navigation also increased the participants’ role identification (feeling of being someone else), which again gave them higher degrees of satisfaction and concert-attending intention. This research contributes to the literature supporting VR’s enhancement of concert experiences and further highlights the important relationship between action, perception, and experience satisfaction.

## Introduction

The coronavirus disease 2019 (COVID-19) pandemic has drastically changed people’s ways of living and working. For example, to avoid physical contact, classes and meetings have been held online. Although technology has made it possible for people to communicate efficiently without physical contact, physical presence still plays an important role for certain social activities, such as attending concerts. Although music performances can now be live-streamed, and the rich, synchronously provided information enables audiences in different places to feel as if the concert were *happening now*, live-streamed performances are still unable to create the feeling of *being there*^1^, which is a key component of concert-going^2^.

### The feeling of being somewhere else: presence

The feeling of *being there* is a key element of *presence*. According to Lee^3^, p. 27, presence is “a psychological state in which virtual (para-authentic or artificial) objects are experienced as actual objects in either sensory or non-sensory ways.” In this study, we focused on the feeling of presence associated with self-location in a mediated environment, which is known as *spatial presence*^4^.

According to Slater and Wilbur^5^, spatial presence is the defining feature of virtual reality (VR), as users can feel that they are in a reality that is virtually created. Therefore, a 2D computer screen can create VR. Another type of VR is mediated via a head-mounted device, with displays mounted close to the eyes producing stereo images that are continuously updated according to the user’s head movements^6^. Compared to 2D computers, head-mounted displays provide users with richer, more authentic sensory information. As Slater and Wilbur^5^ suggested, the degree to which computer displays are capable of producing a vivid illusion of reality can be defined as the *degree of immersion*. Therefore, VR mediated by a head-mounted device produces a more immersive experience than VR mediated by a computer. For simplicity, we use *VR* to refer to the highly immersive VR with the head-mounted device and *computer* to refer to the lowly immersive one. The high-fidelity visual stimulation generated by VR has the potential to cause discomfort, which is known as *cybersickness*^7^. Therefore, any effect observed from the VR device, as opposed to the computer, could be confounded by cybersickness. In this study, we used the Simulator Sickness Questionnaire (SSQ) developed by Kennedy et al*.*^8^ to measure the degree of the participants’ sickness caused by VR, and we used it as a covariate.

The psychology literature has reported substantial evidence of the reciprocal relationship between perception and action^9–11^. Perception affects our actions, and our actions can also shape our perceptions^12^. When one acts toward the environment in a different way, the sense of presence is also likely to change. According to Wei et al*.*^13^, the feeling of control, which refers to a person’s perceived ability to take charge of an interaction with a technology, is a strong predictor of the sense of presence. To put this in more colloquial terms, *doing there* leads to the sense of *being there*^14^. Furthermore, feeling in control has been shown to be a strong predictor of flow experience^15^, satisfaction^16^, and attitudes toward virtual communities^17^.

The sense of presence is a crucial factor in flow experience^18–20^. The concept of flow was first described by Csikszentmihalyi^21^ and refers to a state in which people are highly absorbed in a task without noticing the passage of time and which is generally associated with positive emotions^22–24^. Kim and Ko^19^ found that VR via the head-mounted display elicited a higher degree of flow than did computers when the participants were watching a game. The state of flow can predict the sense of satisfaction^19,25^, which is associated with intention^26^.

To summarize, the sense of presence is a crucial element in concert experience because it can induce the state of flow, which then enhances the sense of satisfaction and behavioral intention. Previous research has shown that the rich and immersive perceptual information offered by VR could be effective in eliciting the sense of presence. Although users’ feeling of control is also a predictor of their sense of presence, how such feeling of control manifests its effects in VR has not been explored yet. As action and perception are interactive, improving users’ feelings of control could amplify VR effects. Another possibility is that since VR already provides users with a highly intensive sense of presence, the feeling of control does not have a strong incremental effect. The last possibility is that VR and the feeling of control independently enhance the sense of presence and the concert experience.

Therefore, one purpose of this study was to experimentally test how VR interacts with users’ feelings of control to enhance their sense of presence and thus, their concert experience. In our experiment, we examined the participants’ feeling of control in a concert based on whether they could voluntarily navigate between the performer’s perspective and the audience’s perspective in a concert. Our first hypothesis is as follows:

**H1:** The adoption of VR technology and the user’s navigation freedom jointly elevate the sense of presence, which could improve the state of flow and increase feelings of satisfaction and concert-attending intention.

### The feeling of being someone else: empathy and role identification

VR technology can transport people from one place to another psychologically. This induced feeling of *being somewhere else* has another function: to induce the illusory feeling of *being someone else*, which is associated with empathy and role identification.

Studies have confirmed the close relationship between VR and empathy. For example, in a study by Fonseca and Kraus^27^, the participants watched videos showing the influence of meat consumption on the environment using a VR device or a tablet computer. The participants in the VR group exhibited higher levels of empathy. Archer and Finger^28^ compared the participants’ attitudes toward news articles presented as physical text on the computer screen and in VR and found that the VR group exhibited higher empathy.

Previous studies on VR and empathy focused on how VR can change people’s attitudes by eliciting empathy. However, it is still unknown if empathy plays an important role in recreational activities, such as going to a concert. To investigate this issue, we turned to the literature on the psychology of entertainment and found that the related concept of *role identification* was more commonly addressed than *empathy*. Cohen^29^ argued that role identification performs an important function in the enjoyment of art. When people are reading a novel or watching a TV show, they are imagining themselves in the main role. Regarding playing games, Klimmt et al*.*^30^ argued that compared to traditional media, such as TV, in which audiences build dyadic or parasocial relationships with the characters, video game players undergo a temporal shift in self-perception and identify themselves with the character in the game, which could be termed *monadic* identification. Such a process has been shown to be strongly associated with game enjoyment^31^. As role identification is highly associated with empathy^32^, a reasonable conjecture is that VR can enhance users’ experiences in recreational activities by triggering their empathy and facilitating their role identification process. Whether this effect applies to concert experience has yet to be tested.

Empathy is not a monolithic construct. *Cognitive empathy*^33^ refers to the capacity to know how other people feel, whereas *affective empathy*^34^ refers to the ability to feel other people’s emotions. As in this study, we dwell on the concert context, in which music might not induce emotions as distinctive as those when watching a movie that has a clear storyline, we focus herein on the cognitive domain of empathy.

Lin^35^ showed that actively playing a game leads to a higher degree of role identification than passively watching someone else play the game. Therefore, we incorporated the active participation factor into our research design (as was the case in H1). Our second hypothesis is as follows:

**H2:** The adoption of VR technology and the users’ navigation freedom jointly elevate the users’ cognitive empathy and role identification, thus increasing their feelings of satisfaction and concert-attending intention.

### Memory

As this study focused on VR-mediated concert experience, the main dependent variables were the feeling of satisfaction and the intention to attend a concert. Previous studies on VR-mediated communication, however, mainly focused on VR effects on memory^36–38^. For example, Schöne et al*.*^39^ presented experimental materials via VR or 2D video and found higher rates of successful retrievals and delayed reaction times in the VR condition than in the 2D condition. To interpret the results, the researchers adopted the autobiographical/episodic memory framework. Episodic memories include events from a first-person perspective and their encoding context, whereas autobiographical memories involve more extended networks of events that incorporate self-reflection, emotional evaluation, and semantic processes^40^. Encoding information from a 2D video involves only the episodic memory process, which means that only fragmented episodic events can be retrieved, whereas with VR, information is encoded by means of the autobiographical memory process.

Similar to H1 and H2, memory can be enhanced by VR and active navigation, possibly through interactions. In a study by Brooks^41^, the participants who could control their own movements performed better in a spatial task than those who could only watch other people’s progress. Using a head-mounted VR display, Hahn et al.^42^ found that the active group performed object recognition better than the passive group. Sauzeon et al.^43^ also identified a memory benefit of active navigation in VR.

Active navigation can sometimes negatively impact memory performance in VR. Batella et al.^44^ found that the passive group performed better than the active group in terms of spatial memory in mixed reality. However, Gaunet et al.^45^ found no difference between the active group and the passive group regarding spatial memory. Possibly, the feeling of dizziness induced by VR^7^ could reduce or even reverse the effects of active navigation. Therefore, our third hypothesis is as follows:

**H3:** The adoption of VR technology and the users’ freedom of navigation interact with their effect on memory.

### Research design

**Figure 1:**
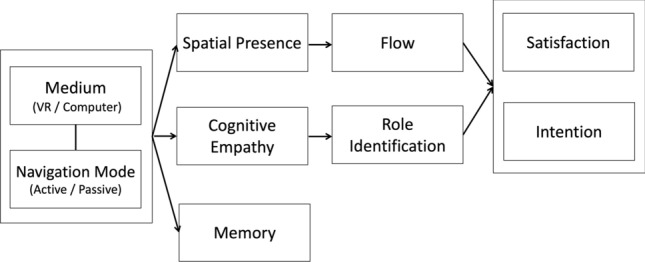
Structure of this study.

Our three hypotheses are integrated and illustrated in Fig. 1. To test them, we conducted an experiment that involved manipulating the medium and the navigation mode.

## Methods

We conducted an experiment in which the medium (VR/computer) and the navigation mode (active/passive) were manipulated as two independent factors. The participants were asked to view a concert video via a head-mounted display, during which they could freely change their perspectives between the audience and the performer in the VR-active condition but not in the VR-passive condition. The same concert video was presented on a computer screen, during which the participants could freely change their perspectives between the audience and the performer in the computer-active condition but not in the computer-passive condition.

### Participants

The protocol of our study was reviewed and approved by the Institutional Review Board B of National Yang Ming Chiao Tung University (#NCTU-REC-109-086E). All the methods were performed following the relevant guidelines and regulations laid down by the ethics committee.

We used G*Power 3^46^ to calculate the required sample size. An earlier study^47^ found that the medium and the navigation mode had an interaction effect of *η*^2^ = 0.12 on the intention to visit a virtual gallery. Based on this effect size, the required sample size was estimated to be 80 to achieve a power of 0.9. We recruited slightly more participants to avoid data attrition. Finally, 88 participants were recruited (39 male, 48 female, and 1 other gender; age: mean = 22.6, SD = 2.45).

### Procedure

Each participant was given a brief explanation of the procedure and was asked to voluntarily sign a consent form. The participant’s task comprised three phases: the pre-experimental phase, the experimental phase, and the post-experimental phase.

#### Pre-experimental phase

Each of the participants was provided a computer to fill out a questionnaire on the SurveyCake platform. The first part of the questionnaire (Supplementary Information) comprised demographic questions on gender and age, and 28 items from the Interpersonal Reactivity Index (IRI) scale^48^ that measured the participant’s trait empathy.

#### Experimental phase

**Figure 2:**
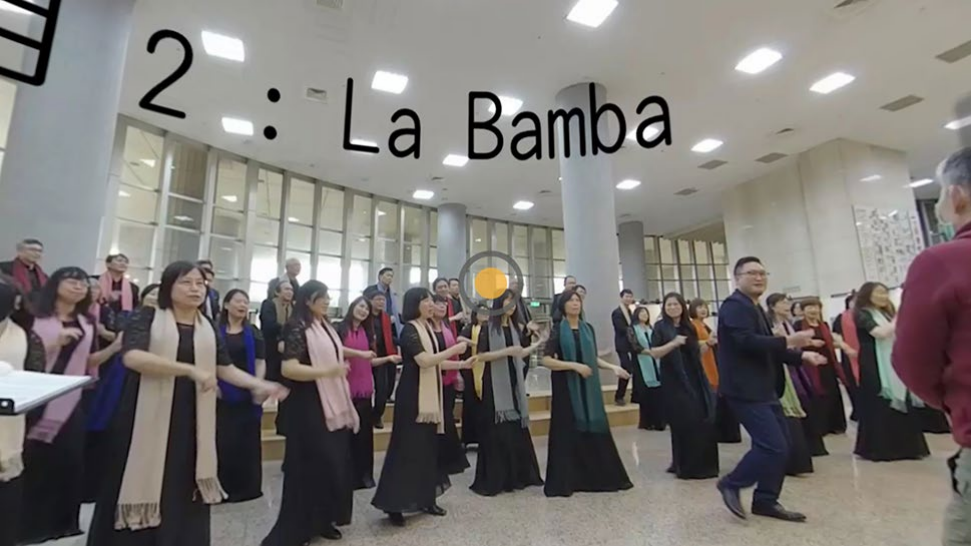
An image from the audience’s perspective.

**Figure 3:**
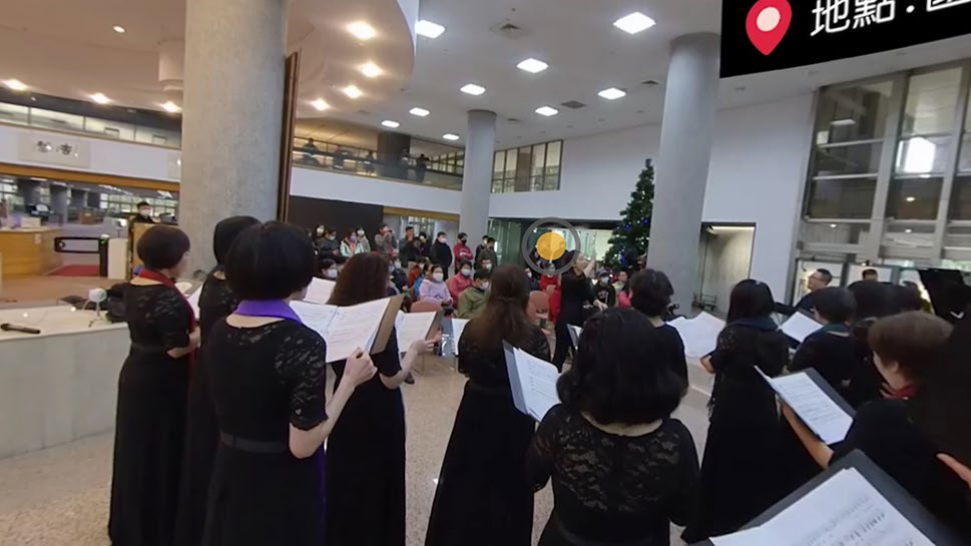
An image from the performer’s perspective.

After answering the questions, the participants were presented the experiment materials, which consisted of 5-min-and-35-s video recordings of a concert by the staff and faculty choir of the authors’ institution. The choir performed two pieces of music: *Bésame Mucho* and *La Bamba*. The videos were recorded using two VR 360 cameras. One camera was placed in the audience area to record the audience’s perspective (Fig. 2), and the other camera was placed on the stage to record the performer’s perspective (Fig. 3).

The participants were randomly assigned to one of four conditions: VR-active, VR-passive, computer-active, or computer-passive. The sound was played via an external loudspeaker connected wirelessly to the head-mounted VR device and the computer.

##### VR-active condition

The participants were asked to put on a head-mounted display. The devices used were Oculus Quest and Oculus Quest 2. Due to the limited availability of both devices, the first seven participants in this condition were asked to use Oculus Quest, and the rest, Oculus Quest 2. The Oculus head-mounted display is an all-in-one device that can track a user’s body and head movements without the need to be connected to another computer. The VR headset functioned such that when the participants turned their head, the view in the headset changed correspondingly.

The experimental video contained video clips of the concert from the audience’s perspective and the performer’s perspective, and the participants could freely change their perspectives by pressing a yellow button (Figs. 2 and 3) using the controller of the VR device.

##### VR-passive condition

As in the VR-active condition, the participants in this condition were also asked to put on a head-mounted display. As in the VR-active condition, the first seven participants were asked to use Oculus Quest, and the rest, Oculus Quest 2. The participants also viewed the video clips from the two perspectives, but the switch between the two perspectives was predefined. The video began with the audience’s perspective, and then switched between the audience’s perspective and the performer’s perspective at the following times (min:s): 1:08, 1:49, 3:24, 4:15, 4:43, and 5:11.

##### Computer-active condition

The participants were asked to watch the concert video clips on a computer with a 22-inch screen and a 1920 × 1080 resolution at a viewing distance of approximately 40 cm. The participants used a mouse to freely change between the audience’s perspective and the performer’s perspective.

##### Computer-passive condition

The participants were asked to watch the concert video clips on a 2D computer screen without the freedom to change their perspectives, as in the VR-passive condition.

Before commencing the experiment, the participants were given a practice session. During the session, they viewed a baritone soloist performing for a few seconds. The participants in the active groups (VR-active and computer-active) were encouraged to press the yellow button to change their practice perspective.

#### Post-experimental phase

After exposure to the audiovisual stimuli of the concert, the participants were asked to return to the computer to fill out the questionnaire (Supplementary Information) that had the following sections.

##### Cybersickness measurement

We used the SSQ^8^ to measure the participants’ degree of cybersickness, and we used the SSQ score as a covariate in the data analysis. The items were measured on a 4-point Likert scale, coded as 0, 1, 2, and 3. We followed the scoring method suggested by Kennedy et al*.*^8^. The 16-item questionnaire comprised the nausea, oculomotor, and disorientation subscales, and we used the total severity score as the cybersickness index. The total severity score was derived by summing the scores for the nausea, oculomotor, and disorientation subscales at the following weights: 1, 1, and 3.74, respectively.

##### Volume

The sound was played via a loudspeaker. To ensure that the sound played in the VR and that played on the computer would have the same volume, we measured the volume and tried to keep its loudness level below 80 dB. Furthermore, we asked the participants to rate, on a 7-point Likert scale, how loud they thought the sound was.

##### Memory

Eighteen multiple-choice questions were formulated to measure the participants’ memories of the concert. The questions encompassed information on the conductor, the performers, the composers of the pieces, and the choir. Two or four response options were provided for each question, but only one of them was correct.

##### Spatial presence

Twelve items were selected from Vorderer et al.^49^, but we changed the word *presentation* to *concert* to suit our study context. The responses were measured on a 7-point Likert scale.

##### Cognitive empathy

Eight items were selected from Shen^50^, but we changed the word *character* to *the performer* or *an audience member* to measure separately cognitive empathy for the performer (four items) and for the audience (four items). The responses were measured on a 7-point Likert scale.

##### Role identification

Fourteen items were selected from Lin^51^ to measure role identification with the performer (7 items) and with the audience (7 items). The responses were measured on a 7-point Likert scale.

##### Flow experience

Six items were selected from Kim and Ko^19^. The responses were measured on a 7-point Likert scale.

##### Satisfaction

Two questions were formulated to measure the participants’ satisfaction levels. The responses were measured on a 7-point Likert scale.

##### Intention

Two questions were crafted to measure the participants’ intentions to attend another virtual concert in the future. The responses were measured on a 7-point Likert scale.

The entire experiment took approximately 40 min, after which each participant was given a gift voucher worth 100 NTD as compensation.

## Results

### Reliability

Most of the critical constructs in this study were measured with multiple items. We first used Cronbach’s alpha to examine whether the multiple items provided a reliable index. The Cronbach’s alpha values were 0.91 for spatial presence, 0.87 for cognitive empathy for the audience, 0.93 for cognitive empathy for the performer, 0.83 for role identification with the audience, 0.86 for role identification with the performer, and 0.94 for flow experience. The correlation coefficient for the two questions on satisfaction was 0.85, and for the two questions that measured intention, 0.86. The internal reliability of the multi-item constructs was generally satisfactory.

The outcome variable of *satisfaction* refers to the audience’s degree of satisfaction with the concert, whereas *intention* refers to the audience’s intention to attend a concert of a similar type in the future. Presumably, these two variables should be highly related. Indeed, when the items from the two variables were combined, their overall Cronbach’s alpha value was 0.88, which was higher than the corresponding value of either of the two variables. To offer a more reliable index of concert experience, we combine the two variables in the following analysis.

### Control variables

Our experiment involved several variables that could potentially affect our data interpretation. The first of these variables was the degree of cybersickness. We analyzed the SSQ scores under different conditions. Presumably, a two-way analysis of variance (ANOVA) should have been conducted, with the two factors being the medium (VR or a computer) and the navigation mode (active or passive), but the assumption of homogeneity of variance was violated [*F*(3, 84) = 10.04, *p* < 0.001]. Therefore, we conducted separate one-way Welch’s ANOVAs, whereby the assumption of homogeneity of variance was not required. For the medium, VR induced a significantly higher degree of cybersickness than the computer [*F*(1, 74.95) = 7.88, *p* = 0.006, *η*^2^ = 0.08], but the effect of the navigation mode [*F*(1, 83.8) = 0.08, *p* = 0.78, *η*^2^ = 0.001] was insignificant. Due to the potential confounding effect of cybersickness, we treated the SSQ score as a covariate in the following analyses.

We also measured the subjective ratings of the sound volume, which likewise violated the assumption of homogeneity of variance [*F*(3, 84) = 3.37, *p* = 0.02]. Therefore, we also conducted separate one-way Welch’s ANOVAs. Both the medium and the navigation mode had no significant effect [*F*(1, 66.86) = 2.14, *p* = 0.15, *η*^2^ = 0.02 and *F*(1, 84.49) < 0.001, *p* > 0.99, *η*^2^ < 0.001, respectively].

### Hypothesis testing

To test each of our three hypotheses, we conducted two-way analyses of covariance (ANCOVA). The two factors were the medium and navigation mode, and the covariate was the participants’ perceived sickness, measured by the SSQ. The dependent variables were spatial presence (H1), cognitive empathy (H2), and memory (H3). Following the ANCOVA, we conducted a mediation analysis to test whether the effects of the medium and the navigation mode could be extended further to satisfaction and concert-attending intention, as illustrated in Fig. 1.

#### Testing H1: presence and flow

Levene's test showed that the assumption of homogeneity of variance was met [*F*(3, 84) = 0.60, *p* = 0.62] for the dependent variable of spatial presence. The medium had a significant effect on spatial presence [*F*(1, 83) = 15.68, *p* < 0.001, *η*^2^ = 0.16] due to the higher spatial presence in the VR condition (*M* = 4.68) than in the computer condition (*M* = 3.98). The navigation mode also had a significant effect on spatial presence [*F*(1, 83) = 14.16, *p* < 0.001, *η*^2^ = 0.15] due to the higher spatial presence in the active group (*M* = 4.67) than in the passive group (*M* = 3.99). The interaction between the medium and the navigation mode was not statistically significant [*F*(1, 83) = 1.42, *p* = 0.24, *η*^2^ = 0.02].

To test the mediation effect mentioned in H1, we used the PROCESS macro^52^ with a bootstrapping method based on 5000 resampling times. Model 6 (double mediation) was selected. As we found separate main effects of the medium and the navigation mode without a significant interaction between them, we used the two factors separately as independent variables to test whether they could affect satisfaction and intention via spatial presence and flow experience. Therefore, the independent variables were the medium (the VR and computer conditions were recoded as 1 and 0, respectively) and the navigation mode (the active and passive conditions were recoded as 1 and 0, respectively); the two mediators were spatial presence and flow; and the outcome variable was satisfaction and intention combined. In the following analyses, a significant mediation effect was indicated by a 95% confidence interval (CI) not crossing 0. The SSQ scores were used as covariates. When the medium was set as the initial independent variable, the mediation effect of spatial presence and flow experience on satisfaction and intention was significant (95% CI [0.17, 0.77]); and when the navigation mode was set as the initial independent variable, the mediation effects of spatial presence and flow experience on satisfaction and intention were also significant (95% CI [0.21, 0.80]). Thus, H1 was supported.

#### Testing H2: empathy and role identification

We conducted an ANCOVA to test the effects of the medium and the navigation mode on cognitive empathy. We added *role* as a within-subject factor because we measured cognitive empathy separately for the performers and the audience. Besides SSQ score, we further added another covariate to the analysis: trait empathy. However, the assumption of homogeneity of variance was not met for the cognitive empathy value from the audience’s perspective [*F*(3, 84) = 3.73, *p* = 0.01] and from the performer’s perspective [*F*(3, 84) = 7.05, *p* < 0.001]. We then chose role identification as the dependent variable, where the assumption of homogeneity of variance was met for both the role identification value from the audience’s perspective [*F*(3, 84) = 0.90, *p* = 0.45] and from the performer’s perspective [*F*(3, 84) = 0.71, *p* = 0.55].

The medium had a significant effect on role identification [*F*(1, 82) = 13.97, *p* < 0.001, *η*^2^ = 0.15] due to the higher role identification in the VR group (*M* = 4.26) than in the computer group (*M* = 3.73). The navigation mode also had a significant effect [*F*(1, 82) = 15.78, *p* < 0.001, *η*^2^ = 0.16] due to the higher role identification in the active group (*M* = 4.37) than in the passive group (*M* = 3.62). The medium and the navigation mode had no significant interaction [*F*(1, 82) = 0.28, *p* = 0.60, *η*^2^ = 0.003]. The role (audience or performer) did not show any statistically significant effect nor interaction with any of the other factors (*ps* > 0.05). Therefore, the rating values of cognitive empathy and role identification for the performer and the audience were combined for the following analysis.

To test the mediation effect mentioned in H2, we used Model 6 (double mediation) of the PROCESS macro, with the SSQ scores and trait empathy included as covariates. The independent variables were the medium (the VR and computer conditions were recoded as 1 and 0, respectively) and the navigation mode (the active and passive conditions were recoded as 1 and 0, respectively); the two mediators were cognitive empathy and role identification; and the outcome variable was satisfaction and intention combined. When the medium was set as the initial independent variable, the mediation effects of cognitive empathy and role identification on satisfaction and intention were significant (95% CI [0.0003, 0.13]) but small. In contrast, the effect sizes were larger when only cognitive empathy (95% CI [0.02, 0.30]) or role identification (95% CI [0.007, 0.26]) was the sole mediator. When the navigation mode was set as the initial independent variable, the mediation effect of cognitive empathy and role identification on satisfaction and intention was insignificant (95% CI [− 0.02, 0.16]). However, the mediating effect was significant when role identification was the sole mediator (95% CI [0.09, 0.45]) but not when cognitive empathy was the sole mediator (95% CI [− 0.03, 0.23]). Thus, H2 was only partially supported. To offer a parsimonious explanation of the mediation effects, we separated cognitive empathy and role identification as two independent mediators.

#### Testing H3: memory

Levene’s test showed that the assumption of homogeneity of variance was violated [*F*(3, 84) = 2.73, *p* = 0.049] for the accuracy of memory. We then conducted a one-way Welch’s ANOVA separately for the medium and the navigation mode but found no significant effect of the medium [*F*(1, 84.55) = 0.95, *p* = 0.33, *η*^2^ = 0.01] and the navigation mode [*F*(1, 78.62) = 1.49, *p* = 0.23, *η*^2^ = 0.02]. To explore a potential interaction between the medium and the navigation mode, we conducted a one-way Welch’s ANOVA on the effect of the condition (VR-active, VR-passive, computer-active, and computer-passive) but found no significant effect [*F*(3, 46.01) = 0.79, *p* = 0.51, *η*^2^ = 0.03]. As we could not include a covariate in the Welch’s ANOVA, we also examined the effect of cybersickness on memory performance and found no significant correlation between them [*r* = − 0.12, *t*(86) = − 1.15, *p* = 0.25]. Therefore, the lack of a significant effect of the medium on memory was unlikely due to the confounding effect of cybersickness. Thus, H3 was not supported. The mean memory accuracy values were 0.67, 0.65, 0.66, and 0.62 in the VR-active, VR-passive, computer-active, and computer-passive conditions, respectively.

## Discussion

**Figure 4:**
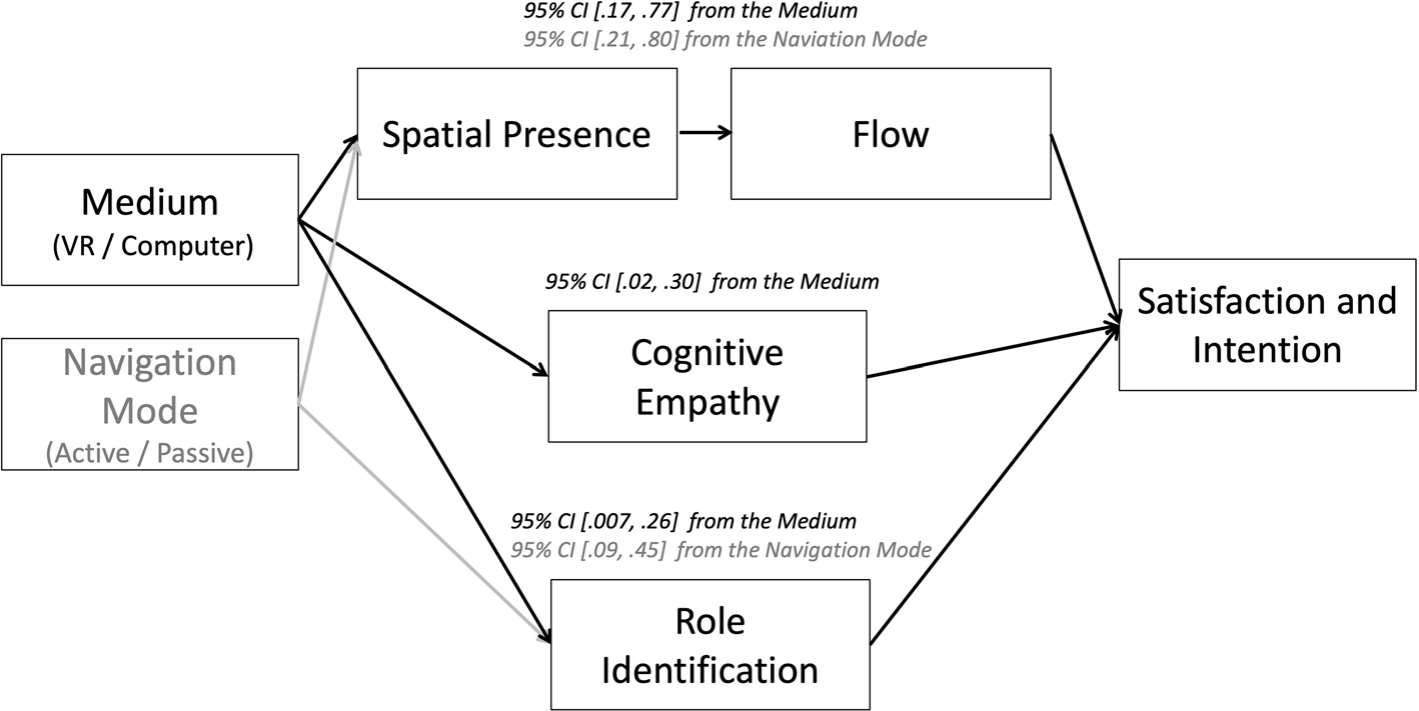
Effects of the medium and the navigation mode. The 95% CI here indicates the 95% confidence interval of the mediation effects, with the medium (marked in black) or the navigation mode (marked in gray) being the initial independent variable.

We conducted the experiment to examine the effects of the medium and the navigation mode on satisfaction, intention, and memory, with spatial presence, flow, cognitive empathy, and role identification acting as potential mediators (see Fig. 1). The test for H1 showed that both VR and active navigation could increase satisfaction and intention by increasing spatial presence and flow. The test for H2 showed that VR can increase satisfaction and intention by increasing cognitive empathy and role identification, but the effect of the active mode was seen only when role identification was the mediator. The test for H3 showed that neither VR nor the active mode boosts memory performance. The results are illustrated in Fig. 4.

### Additive effects of the medium versus the navigation mode

In the cognitive psychology literature, one view of the relationship between perception and action is that they are interactive. According to^53^, how we react to the environment determines how we perceive the environment. This position is also supported by recent findings^9–12^. Compared to the computer, VR provided the audience richer perceptual stimulation that could increase their sense of presence^54,55^, empathy^27,28^, and role identification, as demonstrated in multiple previous studies and in this study. The additive effects of VR and active navigation on both spatial presence and role identification that were observed in this study indicated that the ability to actively interact with the experimental stimuli did not affect the participants’ perceptions of them. The already rich information provided by VR did not seem to become richer when the observer was able to interact with it. Active navigation did help, but not at the perceptual stage; instead, the effect of active navigation was seen after the perception of the scene was complete, which directly increased the participants’ feelings of presence and role identification.

One finding that was inconsistent with our hypotheses was the independence of the mediating effects of cognitive empathy and role identification (Fig. 4). More specifically, we found that higher cognitive empathy due to VR does not guarantee a higher degree of role identification. Our measurement of cognitive empathy was related to whether one can “understand” how others feel, whereas role identification involves the feeling of being someone else. The two constructs are highly related, but our findings suggest that they are not identical. Knowing how it feels to be someone else and feeling what it is like to be someone else are two different abilities mediated by different mechanisms. VR can facilitate both mechanisms, whereas active navigation can facilitate only the latter.

### Concerts and gaming

Klimmt et al.^30^ pointed out that the psychological process involved in playing a video game is qualitatively different from that in traditional entertainment, such as in watching TV. When playing a video game, the players project themselves onto the characters in the game and experience a temporal shift in their self-identities; therefore, the relationship between the player and the character is monadic. When using traditional media, however, such as TV, the relationship between the audience and the characters is dyadic. According to the results of our study, going to a concert can also involve monadic relationships. When the audience was provided rich information or the possibility of active navigation, their role identification increased, which is critical for increasing satisfaction and intention.

This study also helped redefine the distinction between monadic and dyadic relationships in different forms of media. Gaming provides users with a monadic experience in relation to the characters in the media. As technology advances, users may also come to enjoy monadic experiences in other forms of entertainment, such as when attending a concert or watching a movie.

### Lack of effect on memory

We also measured the participants’ memory of the concert, but both the medium and the navigation mode had no effect. A potential flaw in our measurement was that we did not have an index of reliability or validity; therefore, memory could have been affected by our manipulations if our measurement had proper reliability or validity. Nevertheless, we believe that this limitation did not hurt the overall value of our study. In the context of concert attendance, we believe that the audience enjoyment of the performance is more important than how much they can remember. Previous studies on VR benefits or the feeling of control had educational purposes, due to which memory was a critical index. However, when watching a music performance, one’s satisfaction with the performance and one’s likelihood of participating again are more important.

### Cybersickness

Another noteworthy finding was the effect of cybersickness. VR did induce a significantly higher degree of cybersickness than the computer did. Nevertheless, the scores in the computer and VR conditions were 3.68 and 8.57, respectively, which were considered negligible and minimal in the literature^56^. The most common theory regarding cybersickness is sensory mismatch^7^. It states that when different senses perceive the outside environment differently, a mismatch occurs, which causes discomfort. Therefore, cybersickness can be a serious problem if the stimuli in VR are moving while the viewer is not; in this case, the visual stimuli are moving, but the vestibular system receives no motion signal from the viewer’s physical environment. In the context of a concert, the audience and the performer usually do not move, and thus, cybersickness is not a significant problem. Therefore, the concert is actually the perfect context for the adoption of VR. In a virtual concert, VR can exert its positive effects without the users suffering from serious cybersickness.

## Conclusion

We experimentally demonstrated the effects of VR and active navigation. These two features can improve the concert experience by facilitating spatial presence (the feeling of being somewhere else) and role identification (the feeling of being someone else). The elevated sense of presence induced by VR or by active navigation can reduce the difference between a mediated concert and a physical concert. Furthermore, VR and active navigation can provide concertgoers opportunities to experience different perspectives, which physical concerts cannot do.

### Limitations and future studies

In our manipulation of the medium, we used two VR devices due to their limited availability. This might have contributed to the data variability, which might have reduced the effect size. Furthermore, in our manipulation of the navigation mode, the participants could only switch between a particular audience’s perspective and a particular performer’s perspective. Therefore, the visual information that the participants were able to explore was very restricted. Even in the active condition, the participants’ degree of freedom was still far from that in the actual environment. This might have underestimated the effect of the navigation mode. Future studies must enable the participants to experience wider degrees of freedom to navigate in the virtual environment.

## Supplementary Information

Supplementary Information.

## Data Availability

The datasets generated and/or analyzed during this study are available from the corresponding author after reasonable request.
