# Supplementary material for: Investigating how immersive virtual reality and active navigation mediate the experience of virtual concerts
Source: Sci Rep. 2023 May 25;13:8507. doi: 10.1038/s41598-023-35369-0 (PMC10211280; doi:10.1038/s41598-023-35369-0)
Supplement: Supplementary file 1 — Supplementary Information. [file 41598_2023_35369_MOESM1_ESM.pdf]

Appendix

The questionnaire in our study used the following items:

Construct	Item
Demographic questions	• Gender and Age
Trait empathy	• I daydream and fantasize, with some regularity, about things that might happen to me.<br>• I often have tender, concerned feelings for people less fortunate than me.<br>• I sometimes find it difficult to see things from the "other guy's" point of view.<br>• Sometimes I don't feel very sorry for other people when they are having problems.<br>• I really get involved with the feelings of the characters in a novel.<br>• In emergency situations, I feel apprehensive and ill-at-ease.<br>• I am usually objective when I watch a movie or play, and I don't often get completely caught up in it.<br>• I try to look at everybody's side of a disagreement before I make a decision.<br>• When I see someone being taken advantage of, I feel kind of protective towards them.<br>• I sometimes feel helpless when I am in the middle of a very emotional situation.<br>• I sometimes try to understand my friends better by imagining how things look from their perspective.<br>• Becoming extremely involved in a good book or movie is somewhat rare for me.<br>• When I see someone get hurt, I tend to remain calm.<br>• Other people's misfortunes do not usually disturb me a great deal.<br>• If I'm sure I'm right about something, I don't waste much time listening to other people's arguments.<br>• After seeing a play or movie, I have felt as though I were one of the characters.<br>• Being in a tense emotional situation scares me.<br>• When I see someone being treated unfairly, I sometimes don't feel very much pity for them.<br>• I am usually pretty effective in dealing with emergencies.<br>• I am often quite touched by things that I see happen.<br>• I believe that there are two sides to every question and try to look at them both.<br>• I would describe myself as a pretty soft-hearted person.<br>• When I watch a good movie, I can very easily put myself in the place of a leading character.

	• I tend to lose control during emergencies.<br>• When I'm upset at someone, I usually try to "put myself in his shoes" for a while.<br>• When I am reading an interesting story or novel, I imagine how I would feel if the events in the story were happening to me.<br>• When I see someone who badly needs help in an emergency, I go to pieces.<br>• Before criticizing somebody, I try to imagine how I would feel if I were in their place.
Cyber-sickness *(The italic text indicates the subscale.)*	• General discomfort (*nausea, oculomotor*)<br>• Fatigue (*oculomotor*)<br>• Headache (*oculomotor*)<br>• Eyestrain (*oculomotor*)<br>• Difficulty focusing (*oculomotor, disorientation*)<br>• Increased salivation (*nausea*)<br>• Sweating (*nausea*)<br>• Nausea (*nausea, disorientation*)<br>• Difficulty concentrating (*nausea, oculomotor*)<br>• Fullness of head (*disorientation*)<br>• Blurred vision (*oculomotor, disorientation*)<br>• Dizzy (eye open) (*disorientation*)<br>• Dizzy (eye closed) (*disorientation*)<br>• Vertigo (*disorientation*)<br>• Stomach awareness (*nausea*)<br>• Burping (*nausea*)
Volume	How loud do you think the sound was?
Memory	• What is the name of the choir?<br>• Where did this concert take place?<br>• What is the name of the soloist?<br>• What was the name of the first piece?<br>• What is the name of pianist?<br>• What is the name of the conductor?<br>• When did the conductor start leading the choir?<br>• What was the name of the second piece?<br>• Where did the composer of the first piece come from?<br>• Which statement about the first piece is incorrect?<br>• What was the color of the pianist's outfit?<br>• Which statement is incorrect about how the choir performed the first piece?<br>• What is the country of origin for the second piece?<br>• The second piece was written in which language?<br>• Which statement is incorrect about how the choir performed the second piece?<br>• What was the first movement of the second piece?

	• Why did everyone laugh at the end?<br>• Based on the perspective you experienced as a performer, do you think the performer was male or female?
Spatial presence	• I felt like I was a part of the environment in the concert.<br>• I felt like I was actually there in the environment of the concert.<br>• I felt like the objects in the concert surrounded me.<br>• It was as though my true location had shifted into the environment of the concert.<br>• I felt as though I was physically present in the environment of the concert.<br>• It seemed as though I actually took part in the action of the concert.<br>• I had the impression that I could be active in the environment of the concert.<br>• I felt like I could move around among the objects in the concert.<br>• The objects in the concert gave me the feeling that I could do things with them.<br>• I had the impression that I could act in the environment of the concert.<br>• It seemed to me that I could have some effect on things in the concert, as I do in real life.<br>• It seemed to me that I could do whatever I wanted in the environment of the concert.
Cognitive empathy (Audience)	• I can see the audience member’s perspective.<br>• I recognize the audience member situation.<br>• I can understand what the audience member was going through in the concert.<br>• The audience member’s reactions to the situation are understandable.
Cognitive empathy (Performer)	• I can see the performer’s perspective.<br>• I recognize the performer’s situation.<br>• I can understand what the performer was going through in the concert.<br>• The performer’s reactions to the situation are understandable.
Role identification (Audience)	• I felt I was a member in the audience.<br>• I felt the actions of the audience were my actions.<br>• I felt I was the one who made decisions in the concert.<br>• My actions in the concert reflected my thoughts.<br>• My actions in the concert were affected by the context.<br>• I cared about the feedback other people gave to me.<br>• I noticed the consequences of my action in the concert.
Role identification (Performer)	• I felt I was a performer in the concert.<br>• I felt the actions of the performers were my actions.<br>• I felt I was the one who made decisions in the concert.<br>• My actions in the concert reflected my thoughts.

	• My actions in the concert were affected by the context.<br>• I cared about the feedback other people gave to me.<br>• I noticed the consequences of my actions in the concert.
Flow	• I was totally focused on the concert.<br>• I was absorbed intensely.<br>• Time seemed to go by very quickly.<br>• It was enjoyable.<br>• It was exciting.<br>• It was fun.
Satisfaction	• I was satisfied with the content of the concert.<br>• I was satisfied with the experience of the concert.
Intention	• I look forward to going to a virtual concert or a concert conveyed by a VR 360 video in the future.<br>• In the future, I am likely to go to a virtual concert or a concert conveyed by a VR 360 video.
